# Observations on the Superior Advantages of Puncturing Ansarcous Limbs, in Particular States of the Disease, over Other Modes of Treatment

**Published:** 1822-05

**Authors:** 


### THERAPEIA.

**Art. III.**—*Observations on the superior Advantages of puncturing Anasarcous Limbs, in particular States of the Disease, over other Modes of Treatment.* By a Member of the Royal College of Surgeons in London.

MAY I be permitted, through the medium of your invaluable Miscellany, to offer a few observations on the great advantages of puncturing in anasarcous complaints; a practice which, in my opinion, is productive of the most speedy and effectual relief to the patient, when judiciously had recourse to. I am led thus to address you, from the numerous happy results I have lately effected, and which I look upon as my duty to communicate to the profession at large. I shall not trouble you with relating a number of individual cases wherein the operation has been successful; but I shall point out, at once, every characteristic mark whereby, in future, it may be deemed most advisable. It was an operation, at one time, held in great repute: it was a favourite of Galen's, and no doubt, but like many other valuable means, would be indiscriminately resorted to, or be expected to accomplish such miracles as are far beyond the reach of mortal art; till, at last, from its misapplication, it became dangerous, and has gradually dwindled, since, into a state of disregard! My object, however, is not to enter into a minute detail of its history, further than regards the present day. I shall only attempt to establish its former pretensions as a remedy admirably calculated for the relief of certain cases of anasarca:

for there has never yet been any thing which succeeded alike in every one's hands, or gained every one's approbation. In the hands of skilful men, I believe, it will be productive of the most desired success; and will, I trust, prove itself to be of far greater utility in evacuating the fluid diffused through the cellular tissue of the body, than any eulogium I can bestow in favour of its restoration.

The chief reason, perhaps, of this valuable discovery having fallen into disrepute, was the great dread of gangrene after its performance; but, when we take into consideration the manner in which it was performed, we need no longer wonder at this frequent occurrence. In these days, extensive incisions were made, or a number of scarifications adjoining each other, which are equally as improper and dangerous. These were usually about the ankles or feet, or in the most dependent part of the swelling; and many of their incisions were even three or four inches in length, and in deepness in proportion. Although, for the most part, they were followed by bad consequences, yet men were not deterred from their use, from the idea of relieving their patient's sufferings for a time, should they not be so fortunate as to succeed altogether; or that, were the limbs to burst spontaneously, the same dread of gangrene would be unavoidable. These notions, likely, led them into an error much oftener than they would otherwise have been, and caused them to adopt these measures under the most unfavourable circumstances: nay, I dare almost safely venture to assert, that it was seldom, or never, had recourse to, until this very last stage of the disease,—when every other means had failed,—when the skin was so distended as to be almost on the point of bursting,—or when the patients appeared to be most certainly dying; for the great dread of gangrene made it always avoided till the last, a time which, I shall now attempt to show, was the most likely of any for its production.

If the gangrene, which not unfrequently occurs in the toes or extremities of old men, arise, as is generally imagined, from an ossified state of the arterial system, in consequence of which the parts most remote from the heart become deprived of their due supply of blood, may we not, with the same propriety, infer that puncturing in the last stage of diffused dropsy, in an old worn-out constitution, where the circulation has become nearly exhausted,—where the functions are carried on with loss of vigour, and their vitality weakened,—and where we have every reason, also, to expect ossification of the arteries;—will puncturing here, I say, not be almost certain to be productive of the dread in question? The answers to this must decidedly appear in the affirmative, even to the most infatuated capacity? Then it was in similar cases to these, some years ago, that surgeons deemed, chiefly, this operation to be necessary, after they had lavished their whole wonted specifics in endeavouring to renovate the action of the poor languishing absorbents! Moreover, has it never been observed that an anasarca has been a precursory symptom of a casual gangrene of the toes? and has it not, also, been often noticed that a dropsy is as common an attendant on this morbid state of the arteries as any other disease whatsoever? In short, I am inclined to think that anasarcous complaints, occurring in old broken-up constitutions, are almost always dependent upon this change of structure, and would, very generally, advance into a state of mortification, if left to the decision of its own progress, without the surgeon's interference at all. In such cases, the whole body becomes speedily distended; it loses its tone; the skin, no longer able to withstand the increase of pressure, breaks; and sphacelus eventually signs the death-warrant of the sufferer.

[illegible]he greater number of cases which falls under the protection [illegible]e surgeon, are, for the most part, people advanced in years, o[illegible]out the age of fifty and upwards: young subjects are more rarely met with, unless consequent upon some other disease, as scarlet-fever, small-pox, &c. which generally yield, readily enough, to other modes of treatment; or from organic diseases of the heart, or some such malformations of particular parts. In such cases, several remedies may be given with relief; but none, as yet, have been discovered capable of performing a cure.

It is in these instances I should have recourse to the operation of puncturing, as the most speedy mode of affording relief: but, by way of better illustration of my meaning, I shall briefly relate an exemplary case. Suppose I were called to A. B., about twenty years of age, and found him labouring under anasarca of the lower half of the body, in consequence of a palpitation of the heart, of many years' standing; his legs so much swollen as to render him incapable of bending his knees, or of making use of exercise; and his face, arms, and trunk, approaching rapidly into the same state, or perhaps already so; with vehement beating of the heart and arteries, great difficulty of breathing, lividness of the lips and nose, scanty urine; and the swelling, too, not of that leucophlegmatic aspect which characterizes the anasarca in old age, but attended with a very slight degree of floridness; I should surely, without the least hesitation, have immediate access to the operation of puncturing; but not until I had premised it with venesection and some cool neutral purgatives. By so doing, I should expect to accomplish more in one day, than I otherwise should be able to do in a week, were I to rely on the uncertainty of the supertartrate of potash, squills, or even our *great* digitalis. Indeed, I look upon dried squill to be a very useless and inefficacious medicine: in the fresh state it is, by far, more active; and, were I inclined to use it at all, I should certainly give the preference to the latter. It should be given in a liquid form, as it is more apt to affect the stomach and bowels, unless it is given in something like the form of a mixture, as the acetum. In some cases, it has even gone so far as to produce a very violent hypercatharsis; in others, bloody evacuations: therefore, care should be taken not to prescribe it in too large doses at first. Supertartrate of potash has sometimes been of service in carrying off dropsical waters, by its purging effects on the bowels: it answers very well, too, if given at the time the patient is taking other medicine, and particularly in solution as a drink *ad libitum;* but, when taken with a view of promoting a moderate diuresis, in a drachm or half a drachm twice or thrice a-day, it will be found to be a remedy not always to be depended upon. Digitalis does not produce its effects immediately; it is sometimes two or three weeks [illegible] it does so, and then they come on very violent indeed [illegible] a violent vomiting comes on, which is often very difficult to get suppressed: it is sometimes suspended, but returns again, and many fatal accidents have happened from the too free use of this drug. At other times, its effects do not come till a considerable time after it has been withdrawn, perhaps not for four or five days; and then we will notice an increased flow of urine. It will, therefore, be obvious why I prefer puncturing, as early as possible, in such cases as the one I have just alluded to: it is the fear of bringing the patient into an extreme state of weakness, by adopting, unsuccessfully, the above-mentioned remedies, which I look upon would subject him to much greater danger of gangrene at this late period, than when the functions of the body were performing their office in a much more vigorous and active state.

The plan, which I am about to recommend for its performance, is very simple: though I have not generally seen it practised, there is nothing new in it, for it was much recommended by the late eminent Dr. Munro, of Edinburgh; yet, perhaps, its *modus operandi* may not appear to those practitioners who do not adopt it, in the same light in which it has appeared to me.

Before ever I attempt to open an anasarcous limb, I make the patient's friends provide me with a flannel roller; the chief utility of which I shall now attempt to elucidate. It has often struck me with utter surprise at finding patients dead a few days after this operation was resorted to,—patients who had not the appearance of so immediately dying, at the time it was performed. This led me to attempt an investigation of the cause; and, after leading myself into many endless and perplexing difficulties, I, at last, have fancied I have extricated myself by bringing-up to the following conclusions: viz.—That the causes of sudden death may be attributable to the removal of the accumulation of the lymph, which, in the relaxed state of the system at this time, supported the circulation by means of its pressure, similar to a bandage in paracentesis abdominis, or in varicose veins of the leg; instead of the natural muscular tone of the limb and skin, which is now become lost from over-distension. That, when this fluid is evacuated, the pressure necessary for the circulation is also removed; the blood then accumulates in the lower parts and extremities of the body, depriving the heart of its due supply, which already is labouring under a heavy state of exhaustion. The water still continuing to dribble away, still makes room for a further determination of the vital fluid, till at last it becomes so exhausted, from this constant encroachment on its indispensable stimulus, that it becomes no longer able to carry on its function.

Whether this suggestion of mine be correct or not, I cannot pretend to determine: I can only say, that, since I used bandages as a substitute for the pressure, I have succeeded in many cases I think I should have lost before, had I attempted its performance without this precaution. It is not of much importance whether we apply it before, or immediately after, the operation. I generally prefer the latter. I make a puncture, with a common bleeding lancet, on the inner side of the gastrocnemius muscle, a little below the knee, about four or five inches, in both legs; after which, I directly set about applying the bandage, beginning about an inch above the puncture, and carrying it as high up in the groin as I possibly can; giving directions, at the same time, to the patient to tighten it again as often as it becomes necessary, should it become slackened in my absence. It assists the skin in recovering its tone, and serves to enable the absorbent vessels to do their office of taking up the fluid more readily; and, by its firmly embracing the limb, it probably may further tend to diminish the secretion thrown out by the exhalent vessels.

It is highly necessary, after we have performed the operation, that we should apply a piece of adhesive-plaster over the opening, with a hole in its centre, not larger than the puncture itself, by which means the water may be allowed free vent; by neglecting to do which, inflammation will be more likely to ensue, from the constant flowing of the water over the edges of the orifice. As every other species of wound is, more or less, attended with inflammation a few hours after it has been inflicted, so is it the case with anasarcous wounds; but the latter is more peculiarly liable to have it extended, in consequence of the reasons already stated. Should it, however, arise to any extent, we will find the aq. litharg. acet. co., or the besmearing of the part with some mild melted unguent, which gradually hardens as it becomes cold, to be very useful applications.

After the first tension is taken off, we should give the medicines we place most our confidence in; and I know of none better to make a trial of, than fl. ʒj. of the tincture of digitalis, in fl. ʒiij. of the spiritus æther. nitrici. However, it is left to the practitioner's option what medicines to prescribe, the question being quite foreign to the object of this paper.

We ought not to confine our bandaging to the extremities alone, but apply them to the trunk, if necessary, likewise; for sometimes a considerable quantity of fluid is lodged in this part, and, when it is so, bandages are equally as much indicated here as in other parts of the body. To be brief: bandages are of infinite service in every case of anasarcous swellings, whether in old or young subjects, or whether puncturing has been deemed necessary or not. When properly applied, they become a source of comfort to the individual; that is, when they are applied with a gentle degree of uniform pressure throughout the whole course of the swelling: when improperly, they commonly produce aggravation of symptoms, if not of danger. Care should be taken to examine the nature of the swelling, and particularly the skin, before we attempt to apply them; and, if it is one of those attendant on declining years, the slightest pressure will be sufficient to commence with. In young subjects, on the other hand, we may generally venture to apply them with a greater degree of firmness. That flannel bandaging is of the utmost importance in such cases, is certain; for I have seen several instances cured by their application, when nothing else was used but occasionally attending to the state of the bowels.

With respect to people about the age of fifty and upwards, more discernment will be required on the part of the surgeon; for, at this period of life, we certainly run greater risks of bringing on sloughing;* yet, in the generality of cases, it may be resorted to with perfect freedom. The only danger to be apprehended is, delaying the operation too late, or till the skin appears ready to burst of itself. I am persuaded we will stand a far better chance of curing our patient, were we to make our puncture as early as possible, instead of leaving it as the last resource, as has hitherto been so often practised: if we do

---

* We are led to understand, from a late publication, that the arteries of almost all persons, about the age of fifty, are more or less ossified. How far this opinion of Mr. SHAW's be correct, further experience must confirm: it is one, however, I am not inclined to believe, at present, upon such grounds as Mr. S. has adopted. Subjects which are brought into a dissecting-room, are the worst of any for substantiating such an hypothesis.

so, let us prepare ourselves to encounter the most grievous danger. But, now and then, surgeons are not sent for till the patient is actually gasping for breath, unable either to lie or sit in bed, and his face almost purple. Many of these, I have no doubt, have been mistaken for water in the chest, when, perhaps, with more propriety, the greater number of them might have been denominated *water in the lungs*; for, when the whole body becomes distended with water, it is surely not to be expected the cellular tissue of the lungs should remain more exempt than the rest, and which is sufficient at once to account for the lividness of the face and the inability of breathing. In such cases, I should by all means advise the surgeon to puncture, if there be no existing circumstances in the way to forbid it; for I have seen patients derive almost immediate relief from this, beyond the power of comprehension: and, at any rate, no harm can ensue, for they must inevitably die, if trusted longer to the powers of medicine, or if left to the fate of nature. I should recommend all medicine to be withdrawn, after it has been performed; and, as a substitute, advise wine-negus, or Holland's-gin and water. When the fluid has been so far discharged that the body has regained a share of its former tone, I should, then, if it was thought necessary, have again recourse to the aid of medicine, as I have before mentioned; but not till then.

In my conclusion, I should wish to ask any candid man whether he has ever discovered a certain remedy, capable of evacuating a couple of gallons of dropsical liquid out of the body in twenty-four hours, or not? If he fairly confesses he has not, I should advise him to make trial of this operation in his next case, and then he will accomplish it at once, with ease and expedition. Nay, a man must be horribly obstinate indeed, who would doubt such a plan, when nature herself adopts it for the relief of human misery. Has he never heard an old woman say, that once in her life she had had dropsical swellings of her legs,—that her legs burst, run a great quantity of water,—then got well, without the aid of medicine, excepting a dose of salts occasionally, or an aloes pill? So obvious must be the good effects of this important operation to every unbiassed man, that I am surprised it has not become more usually in vogue: but medical men, of late, have become so much attached to the inward virtues of physic alone, that many, I often think, place as implicit faith in working whatever effects they wish upon the body, as a joiner would assume to himself in fixing on the leg of a chair.

It will now appear, Mr. Editor, from the evidence already advanced in favour of the puncturing system, that, under certain conditions, it is the most valuable means of any we are possessed of, and may be resorted to with the utmost impunity; that, in young subjects labouring under anasarca, particularly from diseases of the heart, it is the most desirable mode of treatment; that, in middle age, it is perfectly safe, when performed early, and highly advantageous as soon as ever our most favourite remedy proves unavailing; that, in old age, it is commendable, and that it not unfrequently is attended with surprising success; but that, in extreme old age, on no account whatsoever should it be attempted; or even in people on the verge of fifty who are labouring under great universal debility, or whose arteries are suspected to be in a state of ossification; that in these and other instances, when puncturing cannot be attempted with apparent safety, bandages alone must be used; that we should never puncture without applying a bandage before-hand, or immediately afterwards; and that, where the tone of the extremities is lost, from over-distension, it will be better to suspend the use of every medicine for a few days after the puncturing, and allow the patients a little wine, Hollands, or some other cordial.

*Newcastle-upon-Tyne; March* 12*th*, 1822.

